# DNA Methylation Difference between Female and Male Ussuri Catfish (*Pseudobagrus ussuriensis*) in Brain and Gonad Tissues

**DOI:** 10.3390/life12060874

**Published:** 2022-06-10

**Authors:** Pei Li, Jian Chen, Chuankun Zhu, Zhengjun Pan, Qing Li, Huijie Wei, Guiying Wang, Weiwei Cheng, Beide Fu, Yanhong Sun

**Affiliations:** 1 Fisheries Research Institute, Wuhan Academy of Agricultural Sciences, Wuhan 430207, China; garfieldlp@163.com (P.L.); chenjian.316@163.com (J.C.); xfsckj@163.com (Q.L.); weihuijie2022@163.com (H.W.); whxfsckj@163.com (G.W.); chengweiwei9922@163.com (W.C.); 2 Jiangsu Key Laboratory for Eco-Agriculture Biotechnology around Hongze Lake, Jiangsu Collaborative Innovation Center of Regional Modern Agriculture & Environmental Protection, Jiangsu Engineering Laboratory for Breeding of Special Aquatic Organisms, Huaiyin Normal University, Huaian 223300, China; zhuchuankun@hytc.edu.cn (C.Z.); zhengjunpan@163.com (Z.P.); 3 Ruibiao (Wuhan) Biotechnology Co., Ltd., Wuhan 430074, China; fubeide@163.com

**Keywords:** methylation difference, brain and gonad, female and male

## Abstract

DNA methylation has been found to be involved in sex determination and differentiation in many aquaculture species. The Ussuri catfish (*Pseudobagrus ussuriensis*) is a popular aquaculture fish in China with high economic value in which male-biased sex dimorphism was observed in terms of body size and body weight. In this study, DNA methylation-sensitive RAD sequencing (Methyl-RAD) was used to explore the epigenetic difference between adult male and female samples in brain and gonad tissues. In brain tissues, 5,442,496 methylated cytosine sites were found and 9.94% of these sites were from symmetric CCGG or CCWGG sites. Among these sites, 321 differential DNA methylation sites (DMSs) in 171 genes were identified, while in gonad tissues, 4,043,053 methylated cytosines sites were found in total and 11.70% of them were from CCGG or CCWGG. Among these sites, 78 differential DNA methylation sites were found which were located in 64 genes. We also found several sex-determination genes among these differential methylated genes, such as *amh*, *gsdf* and *hsd11b2* in brain tissues and *slco3a1*, *socs2* and *trim47* in gonad tissues. These results provided evidence for understanding the function of DNA methylation in the sex differentiation in *Pseudobagrus ussuriensis*, which further deepens the relationship between gene regulation and epigenetics.

## 1. Introduction

DNA methylation has been found to play various key roles in animal sex-determination and gonad differentiation by regulating gene expressions [1,2,3]. It integrates genomic and environmental information to foster the expression of genes to generate a particular sex [4]. In environmental sex determination (ESD) fish, half-tongue sole (*Cynoglossus semilaevis*), a high temperature has been found to increase the DNA methylation level in the promoter of *Cyp19a1a* and *Dmrt*, which leads to the suppression of their expressions, thus inhibiting male development [5]. Besides, high temperature-induced masculinization was also found in Nile tilapia (*Oreochromis niloticus*) and both female and male tilapia showed an increase in DNA methylation across the whole genome level after high temperature induction [6]. In genetic sex determination (GSD) species, promoter methylation in diacylglycerol kinase delta gene (*Dgkd*) has been found to be involved in Pacific oyster (*Crassostrea gigas*) sex determination [7]. *Sry* gene has been found to play a key role in mammal sex determination and its hypomethylation had been reported to promote testis development [8].

In the past decades, many genetic studies have been conducted in fish sex-determination at different levels. The identification of sex-specific chromosomes has been carried out in different fishes [9,10,11,12]. For fishes without sex chromosomes, sex-specific markers were conducted in both XX/XY [13,14,15] and ZZ/ZW [16,17] sex determination systems. Besides, sex-specific expressed genes were also identified in olive flounder (*Paralichthys olivaceus*) [18], half-smooth tongue sole (*Cynoglossus semilaevis*) [19] and other species. However, there are only a few studies available on the sex difference between male and female samples in epigenetics in aquaculture species. In Pacific oyster (*Crassostrea gigas*), a whole genome methylation analysis was carried out on female and male gonads which showed a higher level of DNA methylation in male gonads compared with female ones [7]. In tiger pufferfish, 3173 differentially methylated regions (DMRs) were identified between males and females while several differentially methylated genes (DMGs) were also found [20].

The Ussuri catfish *Pseudobagrus ussuriensis* is a popular aquaculture fish in China due to its delicious taste. It has male-biased sexual dimorphism in terms of body size and body weight; therefore, all-male breeding is of great economical value for this species [21]. However, studies about the sex determination of Ussuri catfish are still limited. Zhu et al. found several male-specific markers in this fish, which indicated that Ussuri catfish have a XX/XY sex-determination system [21,22,23]. Pan et al. found 162 sex-related differentially expressed genes in gonad tissues of different sexes in Ussuri catfish [24]. The studies above focused on genomic and transcriptomic differences between female and male Ussuri catfish, but no studies have been conducted on DNA methylation or epigenetic regulation in its sex-determination.

To further improve our understanding about sex differences at the epigenetic level, high-throughput Methyl-RAD was applied to explore methylation difference between different sexes both in brain and gonad tissues. The results of this study will offer useful indications of the role of epigenetic modification in fish sex determination.

## 2. Materials and Methods

### 2.1. Samples’ Preparation

The animal experimental design was conducted in accordance with the guidelines of Wuhan Academy of Agricultural Sciences. In this study, three female (S6, S7 and S8) and three male (S2, S3 and S5) of *Pseudobagrus ussuriensis* samples were used at the age of 2 and were obtained from Fisheries Research Institute, Wuhan Academy of Agricultural Sciences (30°20′ N, 114°14′ E). These fish were reared in pond with a size of 20 m wide by 40 m long and the average depth was about 1.5 m. The average water temperature for the pond was 17.7 °C (4.8–29.7 °C). The sex of these fish was identified by analyzing gametes released. After being anesthetized with MS-222 (SIGMA, St. Louis, MO, USA), the brain and gonad tissues of the fish were collected and snap frozen in liquid nitrogen. The extraction of genomic DNA was performed using a Rapid Animal Genomic DNA Isolation Kit (Sangon, Shanghai, China) following the manufacture’s instructions. The quality of extracted DNA was assessed by agarose electrophoresis while the quantity of DNA was measured using NanoDrop 2000 (Thermo Scientific, Boston, MA, USA). All qualified high-quality DNA was kept frozen at −20 °C for following procedures.

### 2.2. Methyl-RAD Library Construction and Sequencing

Methyl-RAD libraries were constructed for brain and gonad tissues in both male and female samples following the original protocol [25]. Briefly, 300 ng of high quality DNA was digested by 1U FspEI enzyme (NEB, Ipswich, MA, USA) for 4 h and then ligated to adapter 1 and adapter 2 at 4 °C for 8 h. FspEI enzyme can recognize both 5-methylcytosine (5-mC) and 5-hydroxymethylcytosine (5-hmC) in C^m^C and ^m^CDS sites with the help of an activator (S=C or G; D=A or G or T) [26]. Then, the ligation products were amplified with a set of four primers, which introduced a specific barcode for each library. Finally, the amplification products were separated by 8% polyacrylamide gels by electrophoresis (PAGE) at 120 V for 45 min and then a Poly-Gel DNA Extraction Kit (Sangon, China) was used to recollect target fragment. Each library was sequenced using the Illumina Next500 platform with a PE150 module. Raw reads were filtered with in-house Python scripts to remove reads without restriction sites. Filtered reads were mapped to the reference genome with Bowtie2 v2.3.5.1 [27].

### 2.3. Comparation of Methylation Level between Different Groups

After mapping reads to a chromosome-level Ussuri catfish genome Ps.v1.0 with a size of 741.97 Mb (Zhu et al., unpublished data), the methylation sites were identified according to the cytosine position. In brain and gonad tissues, methylation sites detected with no less than three reads in at least three samples were used for the differential DNA methylation analysis [25,28]. The analysis between the female and male group was conducted based on the qCML method within the R package edgeR [29]. Bonferroni correction was used to control the false discovery rate in multiple comparisons [29]. Lastly, differential methylated cytosines which were located in the gene body and their 2 kb upstream or downstream region were kept for differential methylated genes’ analyses.

### 2.4. Enrichment Analysis of Differentially Methylated Genes (DMGs)

To analyze the functional enrichment of differentially methylated genes, Gene Ontology (GO) and Kyoto Encyclopedia of Genes and Genomes (KEGG) pathway enrichment analyses were carried out with GeneSCF v1.1 [30]. A GO enrichment analysis was conducted using the zfin database and *p* < 0.05 indicated significant enrichment by DMGs. A KEGG enrichment analysis was conducted with the dre database and *p* < 0.05 indicated significant enrichment.

### 2.5. Correlation of Gonad DNA Methylation and Gene Expression

To validate whether DMSs in gonads reverse regulates gene expression, transcriptome data of gonads from female and male fry 40 days past hatch were collected from Pan et al. [24]. First, significantly differentially expressed genes (DEG, FDR < 0.05) from male and female gonads were extracted and their sequences were mapped to genome. Then, corresponding gene pairs were established between DEGs and DMSs using Pearson’s test (*p* < 0.05) and fold changes were compared between them.

## 3. Results

### 3.1. In Silico Analysis of FspEI Sites in Pseudobagrus Ussuriensis Genome

In this study, the FspEI enzyme was used to perform reduced methylome sequencing for DNA methylation profiling. In the Ussuri catfish genome, there are 63,634,074 potential FspEI digestion sites with a density of 11.65 bp (genome length 741,956,528). Among those sites, a 32 bp fragment can be generated if it is symmetrically methylated. For the two primary symmetric methylated sites, there are 650,345 CCGG and 1,086,011 CCWGG with genome-wide densities of 1140 bp and 683 bp, respectively, covering 5.15% of total CGs and 3.50% of CHGs (H=A, C and T).

### 3.2. Sequencing of Brain and Gonad Tissues with Methyl-RAD

Six Methyl-RAD libraries for brain tissues and another 6 libraries for gonad tissues in 3 female and 3 male Ussuri catfish were constructed in this study. For brain tissues, a total of 216,149,198 raw reads were obtained with an average of 36.02 million reads per library. The number of raw reads ranged from 26.26 million to 51.63 million. After filtering with low quality reads, about 32.63 million high-quality reads were retained for each library with a filtering ratio of about 90.25% (Table 1). For gonad tissues, a total of 234,654,091 raw reads were generated with an average of 39.10 million reads in each library. The number of raw reads ranged from 21.33 million to 54.19 million. About 35.16 million high quality reads were maintained for each library a filtering ratio of about 89.57% (Table 1).

**Figure 1:**
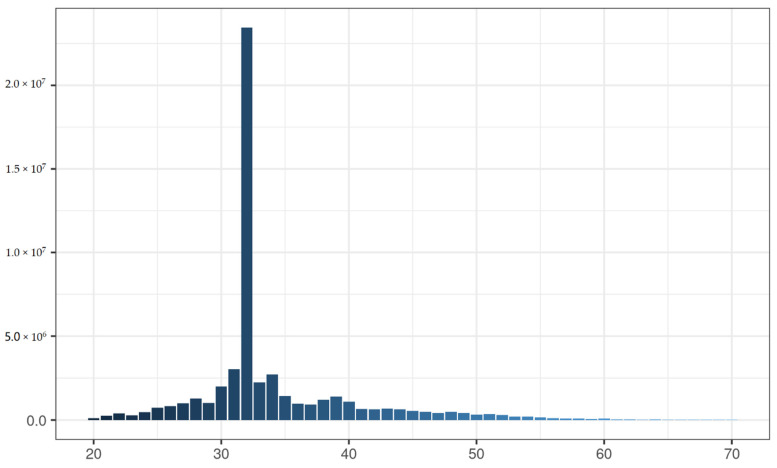
Filtered reads length distribution for MethylRAD sequencing of sample Gonad.S2 from Ussuri catfish (*Pseudobagrus ussuriensis*), which had the most filtered reads in gonad tissues.

As the reads generated by Methyl-RAD may be symmetrical or asymmetrical, their length distribution showed that about 43.36% of all reads in Gonad.S2 were 32 bp, which indicated that they were symmetrically methylated (Figure 1). This pattern is presented in other libraries as well. Additionally, we also found that 25.24% of filtered reads were from C^m^C while the other 21.72% were from CDS (Figure 1).

### 3.3. Identification of Differential Methylation Sites

**Figure 2:**
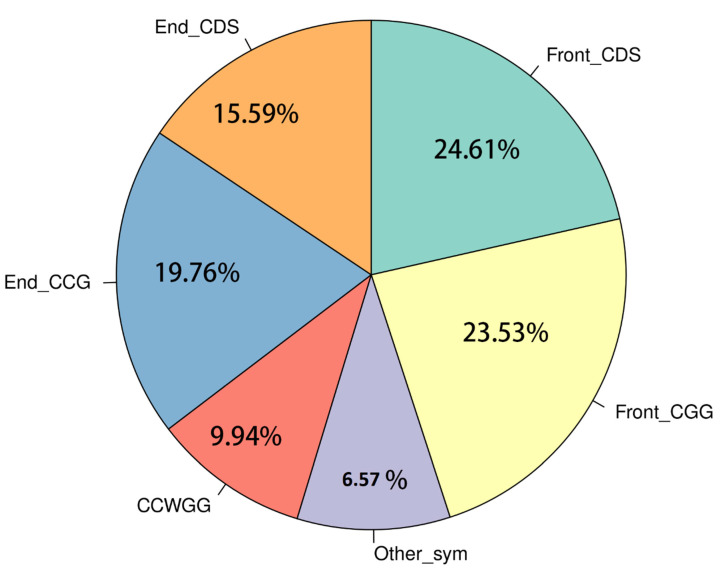
Type of methylation cytosines found in Brain.S7 from Ussuri catfish (*Pseudobagrus ussuriensis*). W = [A/T], D = [G/A/T], S = [G/C]. Front means the enzyme digestion cut was in the 5′ upstream of methylated cytosines. End means the enzyme digestion cut was in the 5′ downstream of methylated cytosines.

**Figure 3:**
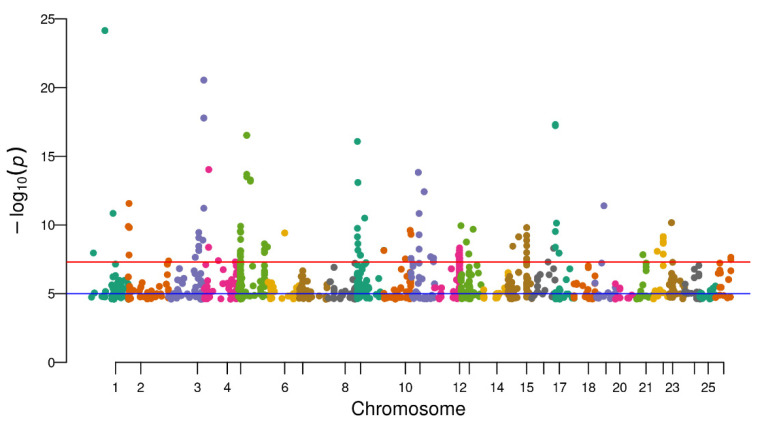
The distribution of 1623 DMSs in brain tissues on 26 chromosomes of Ussuri catfish (*Pseudobagrus ussuriensis)*. Different color corresponding to different chromosomes.

**Figure 4:**
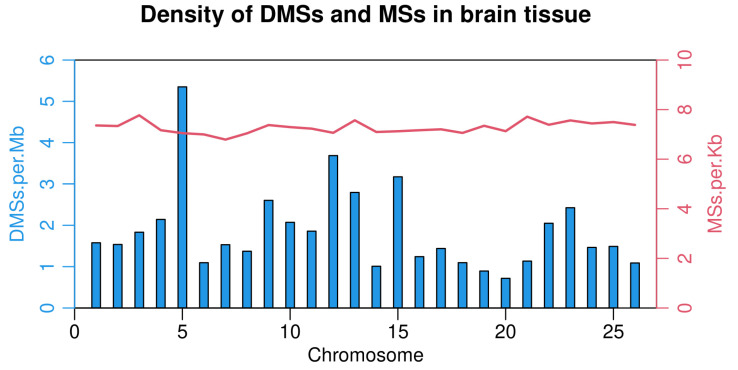
Density of differential methylated sites (DMSs, left) and methylated sites (MSs, right) for each of the 26 chromosomes in brain tissues of Ussuri catfish (*Pseudobagrus ussuriensis*).

After filtering low-quality reads, high-quality reads were mapped to the Ussuri cat fish reference genome, and methylated cytosine sites were identified. In brain tissues, 5,442,496 methylated cytosines were found and 9.94% of these sites belonged to CCGG and CCWGG. Moreover, 40.20% of all methylated cytosines were from ^m^CDS sites and 43.29% were from C^m^CG sites (Figure 2). After filtering the sites which were presented in less than three samples, a total of 3,195,177 sites were used in the differential methylation analysis. PCA analysis showed that male samples were not separated from female samples thoroughly (Supplementary Figure S1). Additionally, 1623 differential DNA methylation sites (DMSs) showed significant differences between the female and male groups (FDR < 0.05). Among these sites, 588 DMSs had a higher methylation level in male and the rest of the 1035 sites had a higher methylation level in females (Supplementary Table S1). Furthermore, these 1623 DMSs were distributed on all 26 chromosomes, among which Chr5 and Chr12 have more DMSs than other chromosomes (Figure 3 and Figure 4).

As DNA methylation in a gene’s promoter region is vital to the regulation of gene expression, we focused on methylated cytosine not only within the gene body but also 2 kb upstream of the transcription start sites (TSS). In total, 1,624,073 methylation sites were identified which were located within 34,269 gene bodies and the promoter region in brain tissues. The median of methylated cytosine for each gene’s promoter region was 10 (Supplementary Figure S2). We also found 321 DMSs in brain tissues and 144 sites had a higher methylation level for females than males (Supplementary Table S2). These 321 DMSs were located in 171 genes, and the enrichment analysis showed that they were involved in methylation (GO:0032259), intracellular signal transduction (GO:003556), phosphorylation (GO:0016310) and the regulation of gene expression (GO:0006355) (Supplementary Table S3). Among the top 100 DMSs with the greatest methylation difference in brain tissues, 20 of them were located in *Rabggtb* (Rab Geranylgeranyltransferase Subunit Beta). Other genes with multiple DMSs were *trioa*(6), *fmnl2b*(6), *mthfd1b*(4) and *hdac11*(4).

In gonad tissues, 4,043,053 methylated cytosine sites were found in total and 2,249,438 were kept for the DMS analysis. A PCA analysis for all six samples showed that the male group and female group were separated well from each other (Supplementary Figure S3). In total, 1923 DMSs were identified between the two groups; 658 DMSs had a higher methylation level in males; and the rest of the 1256 DMSs had a higher methylation level in the female group (Supplementary Table S4). Among all DMSs, 90 were located in Chr09, which is a higher number than for the other chromosomes. Furthermore, 997,280 mCs were identified within 33,950 genes and promoter regions in gonad tissues. Among these mCs, 78 DMSs were located in 64 genes. Among these genes, *trioa*, *snx13*, *ppfibp2b*, *phkb*, *atp13a1* and *actb1* were found to have more than one DMS. Additionally, 10 genes were found among DMGs in both brain and gonad tissues. Among these ten genes, six were unannotated protein-coding genes while the remaining four genes were *tioa*, *atp13a1, atp6v0a2b* and *golgal*.

Using the transcriptome data from previous studies, we re-annotated the *Pseudobagrus ussuriensis* genome, although no protein coding genes were found on the five male-specific sequences [21,24]. Methylated cytosines were found on three scaffolds, scaffold139654, scaffold51227 and scaffold78223 in both tissues, but none of these sites were DMSs.

### 3.4. DMSs within Sex-Related Genes in the Two Tissues

DNA methylation, as well as mRNA expression, varied greatly in different tissues or at different stages of the same tissue. In brain tissues, DMSs were identified in several sex-determination (SD) genes, such as *amh*, *gsdf*, *wt1a*, *wt1b* and *hsd11b2*. However, no DMSs were found in other common SD genes, such as *cyp19a1a*, *foxl2b*, *sox9a*, *wnt4b*, *dmrt1* or *sox8a*. With regard to genes expressed in differentiated gonads, two DMSs were presented in *slco3a1*, one in *socs2* and one in *trim47*, which were associated with male-specific gonad differentiation. For female-specific gonad differentiation genes, two DMSs were observed in *asns*, *dapk1* and *rpgrip1* and one DMS was observed in *ctnnb1*, *actr6* and *smpdl3b* (Table 2). In addition, several DMSs were identified in genes related to high-temperature-induced masculinization in Nile tilapia (*Oreochromis niloticus*) [31]. For example, two DMSs were discovered in *Kdm6bb* and six DMSs in *Jarid2*, both of which were up-regulated in male brains when compared with female brains. Besides, 18 DMSs were presented in heat shock proteins (HSPs) families, such as hspa2, hspa5 and hspa9, which has been reported to act as a steroid receptor and plays an important role in sex differentiation [31]. In gonad tissues, only one DMS was found in *rspol* and one DMS in *dapk*, both of which were female-specific gonad differentiation genes [32]. Besides, one DMS was identified in *sox10* and one DMS in *pdgfra*, which were reported to be involved in sex differentiation in Nile tilapia [33].

### 3.5. Correlation between DNA Methylation and Transcriptome in Gonad Tissues

**Figure 5:**
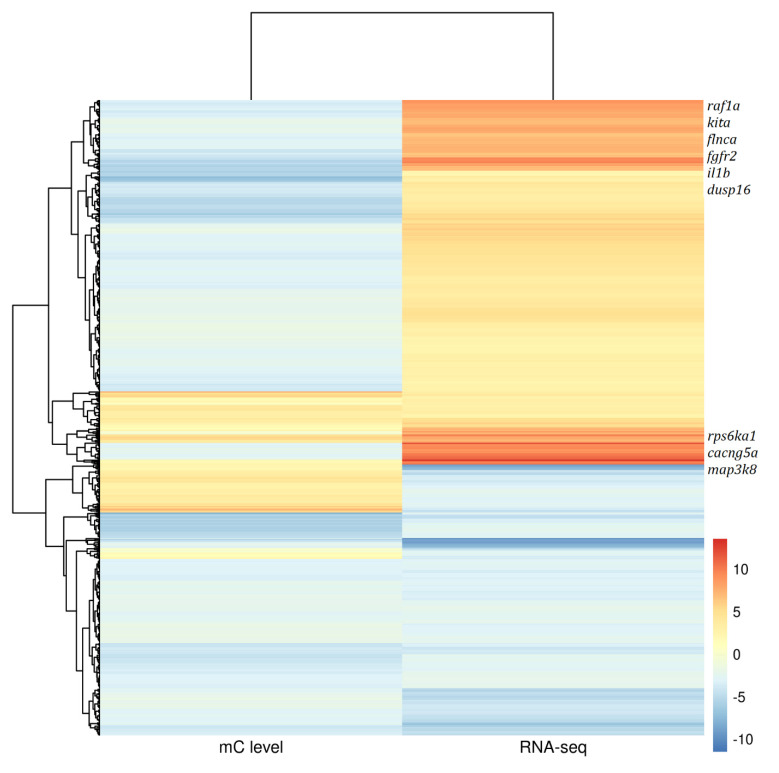
Heatmap for differential methylated genes’ DNA methylation level and their RNA-SEQ gene expression from gonad tissues of Ussuri catfish (*Pseudobagrus ussuriensis*).

Previous studies found a negative correlation between gene methylation and its expression [2,3]. In this study, gonad transcriptome data of *Pseudobagrus ussuriensis* derived from Pan et al. were used to investigate the correlation between DNA methylation and gene expression patterns. First, 673 genes were found to be significantly differentially expressed between female and male gonads and were also apparent in the DNA methylation levels. The correlation between the two datasets was then investigated and 391 genes were found to be negatively correlated (Figure 5). A KEGG enrichment analysis found that these genes were enriched in Oocyte meiosis (dre04114), the MAPK signaling pathway (dre04010), Wnt signaling pathway (dre04310) and GnRH signaling pathway (dre04912) (Supplementary Table S5).

## 4. Discussion

Sex differences in the genetics between males and females has been reported in many aquaculture species in the form of sex-specific markers [13,21,34] or sex-specific gene expression [18,19,24,35]. However, epigenetic difference between sexes have rarely been reported [7]. In this study, DNA methylation-sensitive RAD (MethylRAD) sequencing was conducted to generate the first large-scale, single-base resolution methylomes for male and female *Pseudobagrus ussuriensis.* MethylRAD possesses several advantages when compared with whole genome bisulfite sequencing (WGBS). It does not require the bisulfite conversion of sample DNA, which may cause losses in the epigenetic information for parts of the genome [25,36]. It requires less sample DNA (1–500 ng) than WGBS (1–1.5 ug), which is suitable for the methylome study of precious samples [25]. Lastly, streamlined MethylRAD library construction can be completed within two days and it does not require tedious gel-purification, which makes it ideally suited to large-scale methylation profiling [25].

Whole genome methylation analyses showed no significant difference between male and female brains, and this pattern was also observed when focusing solely on the methylated cytosines located within the gene body and their flanking 2 kb regions. This pattern is consistent with previously published results [37]. However, 1623 DMSs were still detected in brain tissues and 321 of them were located in genes. Additionally, 20 of the top 100 DMSs were located in *Rabggtb*, which is part of geranylgeranyl transferase that transfers alkyl or aryl groups, alongside methyl groups [38]. KEGG enrichment showed that these DMGs were involved in the TGF-beta signaling pathway (ko04350), MAPK signaling pathway (ko04010) and Insulin signaling pathway (ko04910). These results were similar to the DNA methylation difference in queen and honeybee’s brain, which can affect the reproductive and behavior phenotype [39]. Besides, RNA-Seq results from different sexes also proved that the sex difference in brain’s gene expression might contribute to the gonad differentiation in rainbow trout and gar [40,41].

Gonad differentiation is modulated by genes that work in coordination and with precision [42]. In this study, only 78 DMSs were discovered in gonad tissues. This number is much less than that in brain tissues and the reason might be that the samples we used in this study were only taken from fish aged 2 (post hatch). It usually takes 3 years for *Pseudobagrus ussuriensis* to become sex mature, so the gonad tissues used here were still in stage III, meaning they were not yet ready for gamete release. In gonad tissues, 10 methylated cytosines were located in *dmrt1* gene’s promoter region. Although none of them were significantly differentially methylated after multiple tests, we still found their methylation level was higher in male than that in female samples (*p* < 0.05). Combined with the higher expression level of *dmrt1* in male gonads [24], a positive association between the methylation level and gene expression was found for this sex-determination gene. For another key sex-determination gene, 21 methylated cytosines were located in *cyp19a1a*, but none of them showed significant differences in the methylation level between male and female gonads. This phenomenon is different from sea bass and half smooth tongue, both of which share a mixed genetic and environmental sex-determination system [5,43].

The testis has the most complicated expression pattern in comparison to other organs in mammals [44]. Therefore, the methylation level of testis was lower compared to the brain in terms of the methylated cytosines identified. In this study, a higher number of DMSs were found in brain tissue than in gonad tissues. The reason for this phenomenon might be the higher overall methylation level in brain, which led to more methylated cytosines being detected in this study. This phenomenon is in contrast to a similar expression study on hermaphrodite sparid sharpsnout seabream (*Diplodus puntazzo*), which identified a distinct gonad expression pattern and almost the same brain expression pattern between males and females [45].

Many studies investigating the role of DNA methylation on sex determination in animals have been conducted by analyzing methylation level variations in sex determination genes. In red-eared slider turtles (*Trachemys scripta*), methylation decreases in the promoter of the aromatase (*cyp19a1*) were observed when eggs were hatched from a male-producing temperature to a female-producing temperature [46]. *gsdf* has been found to act as male sex initiator in Nile tilapia whose methylation levels lowered at a high-temperature, which induced male sex determination [6]. To dive deeper into this issue, the methylation of 58 documented sex-determination genes was compared between female and male groups in half-tongue sole [5]. Compared with such results, we found a similar pattern of methylation difference for some genes, such as *amh*, *gsdf*, *wnt1a* and *wnt1b*, but no methylation difference was found for other genes both in half-tongue sole and our study, such as *foxl2*, *sox9a* and *wnt4b* [5]. These results indicate that GSD and ESD species share methylation patterns of sex determination genes to a high degree.

## 5. Conclusions

In this study, we used Methyl-RAD to investigate the methylation difference between female and male samples in both brain and gonad tissues. In total, 321 and 64 differentially methylated genes were found in brain and gonad tissues, respectively. These DMGs showed a similar methylation pattern with previously published fishes, which indicated that they shared a common mechanism in terms of the role of DNA methylation in fish sex determination.

## Figures and Tables

**Table 1:** Statistics for all MehtylRAD libraries in brain and gonad tissues.

Sample Name	Sex	Raw Reads	Filtered Reads	Filtering Ratio (%)	Mapped Reads	Mapping Ratio (%)
Brain.S2	Male	40,210,692	35,940,564	89.38	33,617,786	93.54
Brain.S3	Male	27,314,220	24,250,093	88.78	21,873,354	90.20
Brain.S5	Male	26,268,118	22,466,325	85.53	21,099,573	93.92
Brain.S6	Female	43,969,478	41,163,429	93.62	37,713,432	91.62
Brain.S7	Female	51,638,882	47,078,004	91.17	42,662,127	90.62
Brain.S8	Female	26,747,808	24,889,783	93.05	23,506,211	94.44
Gonad.S2	Male	58,312,444	54,198,333	92.94	51,349,811	94.74
Gonad.S3	Male	43,239,451	39,901,859	92.28	37,868,629	94.90
Gonad.S5	Male	34,090,746	30,991,462	90.91	29,176,625	94.14
Gonad.S6	Female	39,853,622	36,158,180	90.73	34,418,333	95.19
Gonad.S7	Female	35,711,187	28,402,596	79.53	26,591,287	93.62
Gonad.S8	Female	23,447,451	21,338,353	91.01	20,085,230	94.13

**Table 2:** Differential methylated cytosines located within gonad differentiation genes of Ussuri catfish (*Pseudobagrus ussuriensis*).

Chr_pos	Brain.S2	Brain.S3	Brain.S5	Brain.S6	Brain.S7	Brain.S8	logFC	logCPM	P Value	NCBI-ID
Chr23_13388333	30	15	6	5	0	4	−2.66536	−0.18741	0.00429	*hsd11b2*
Chr01_21298258	5	6	5	1	2	0	−2.76179	−1.40398	0.005563	*amh*
Chr17_1735529	2	2	3	25	19	7	2.292237	−0.41272	0.008036	*gsdf*
Chr03_6244280	24	12	22	48	285	50	2.108203	2.14866	0.001042	*asns*
Chr07_16130430	63	44	7	12	13	4	−2.35634	0.893175	0.001071	*smpdl3b*
Chr25_9914354	21	20	6	4	2	4	−2.51551	−0.21595	0.002938	*rpgrip1*
Chr03_6241626	26	35	4	7	4	4	−2.46832	0.206087	0.004033	*asns*
Chr18_3386617	20	29	2	6	3	1	−2.75775	−0.14231	0.005502	*ctnnb1*
Chr25_9914339	16	19	7	3	3	5	−2.245	−0.28619	0.006838	*rpgrip1*
Chr01_28622581	0	6	0	32	27	8	2.808632	−0.16436	0.007763	*dapk1*
Chr01_28630117	32	31	3	9	5	4	−2.20926	0.228569	0.008812	*dapk1*
Chr14_6728786	6	4	12	35	40	27	1.713213	0.603745	0.009119	*actr6*
Chr10_13684033	1	0	1	8	29	4	3.584836	−0.83561	0.003608	*slco3a1*
Chr19_8999145	26	20	2	2	5	3	−2.55417	−0.2437	0.005889	*trim47*
Chr20_1768252	9	19	6	5	3	1	−2.38905	−0.53371	0.00796	*socs2*
Chr10_13691493	10	21	2	3	1	2	−2.78154	−0.63388	0.008892	*slco3a1*

## Data Availability

The raw sequencing data were deposited in the NCBI Sequence Read Archive (SRA) under accession numbers PRJNA844593.

## Supplementary Materials

The following supporting information can be downloaded at: https://www.mdpi.com/article/10.3390/life12060874/s1, Table S1: Methylation statics for 1623 DMSs in brain tissues; Table S2: Statistics for 321 DMSs in gene region from brain tissues; Table S3: KEGG enrichment for 321 DMGs in brain tissues. Table S4: Statistics for 1923 DMSs from gonad tissues; Table S5: KEGG enrichment for negative correlated genes between DMSs and DEGs in RNA-SEQ in gonad. Figure S1: Number of mC in gene’s promoter region in brain tissues; Figure S2: The number distribution for methylated cytosines in gene’s promoter region. Figure S3: PCA analysis for all 6 samples in gonad tissues.
