# Supplementary figures and images for: DNA Methylation Difference between Female and Male Ussuri Catfish (Pseudobagrus ussuriensis) in Brain and Gonad Tissues
Source: Life (Basel). 2022 Jun 10;12(6):874. doi: 10.3390/life12060874 (PMC9228513; doi:10.3390/life12060874)

# MDS for mC in promoter region

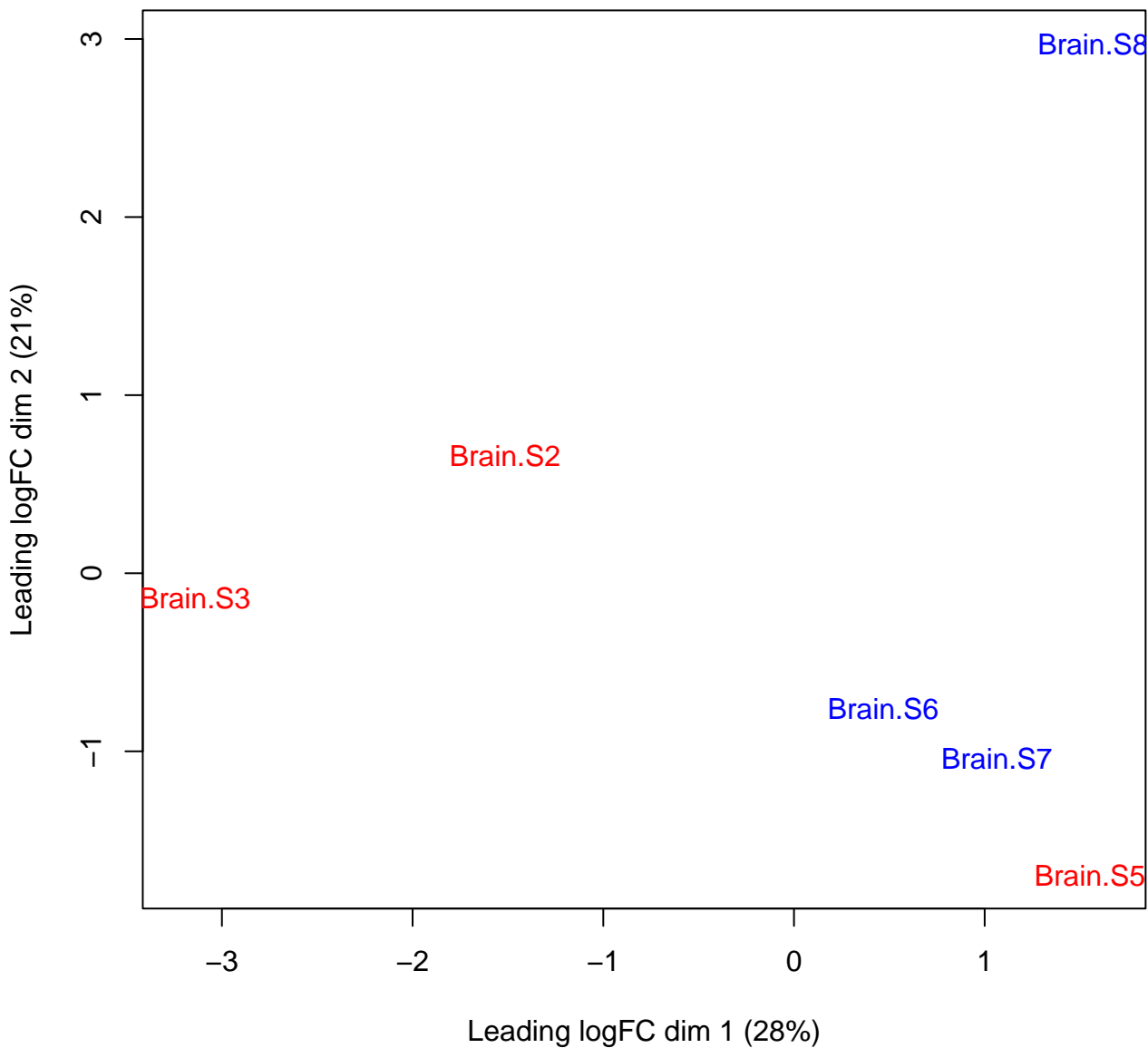

Supplement: Supplementary file 1 [file life-12-00874-s001.zip › Supplementary Figure S1.pdf]

# Number of mC in gene's promoter region

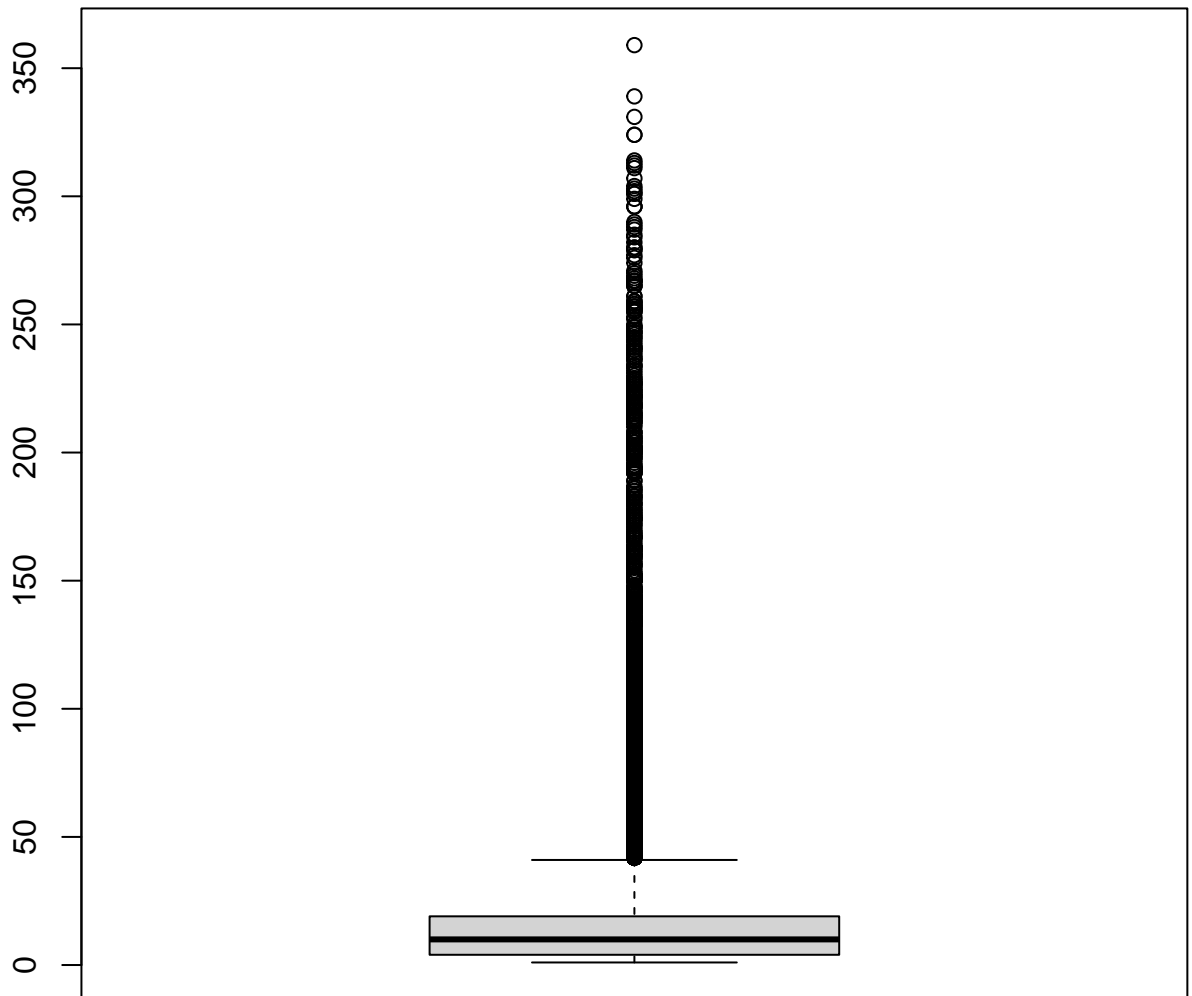

Brain tissue

Supplement: Supplementary file 1 [file life-12-00874-s001.zip › Supplementary Figure S2.pdf]

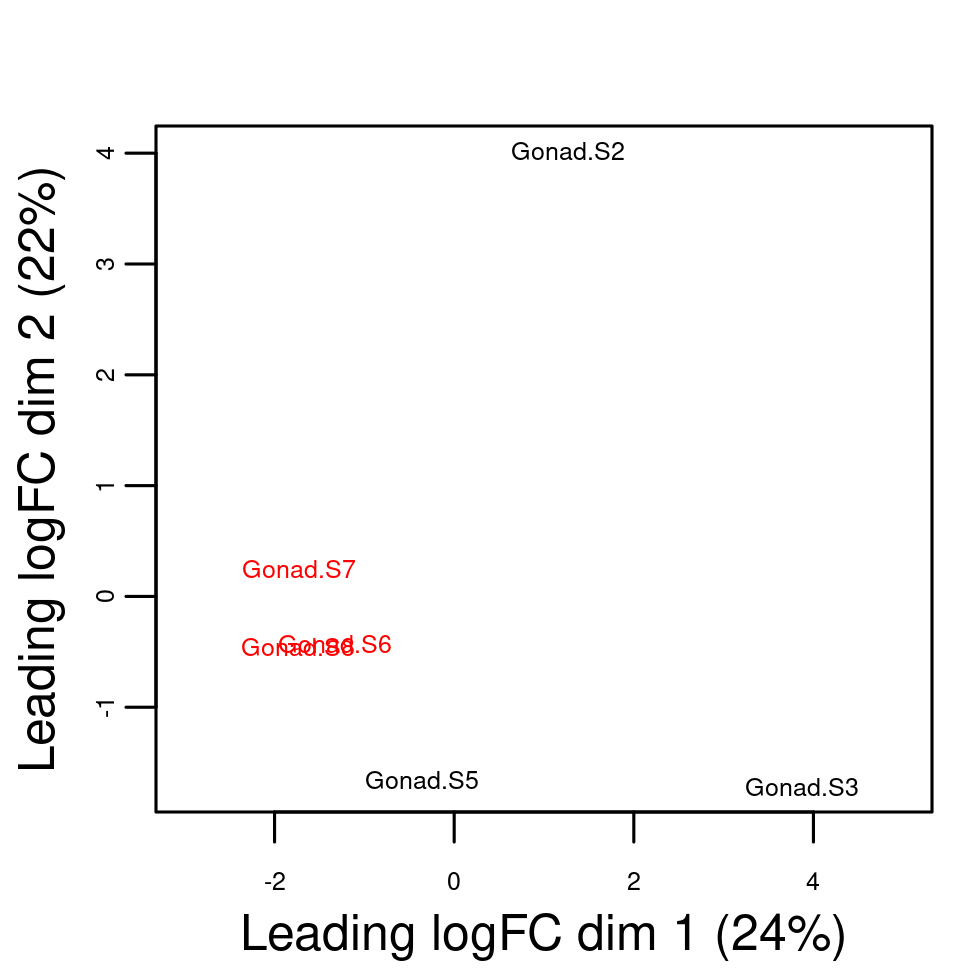

Supplement: Supplementary file 1 [file life-12-00874-s001.zip › Supplementary Figure S3.tif]
